# Efficacy and safety of memantine in patients with moderate-to-severe Alzheimer's disease: results of a pooled analysis of two randomized, double-blind, placebo-controlled trials in Japan

**DOI:** 10.1517/14656566.2014.902446

**Published:** 2014-03-27

**Authors:** Yu Nakamura, Shin Kitamura, Akira Homma, Kazuhito Shiosakai, Daiju Matsui

**Affiliations:** ^a^ Kagawa University, Faculty of Medicine, Department of Neuropsychiatry, 1750-1 Ikenobe, Miki-cho, Kita-gun, Kagawa, Japan+81 87 898 5111; +81 87 891 2016; yunaka@med.kagawa-u.ac.jp; ^b^ Nippon Medical School Musashikosugi Hospital, Department of Internal Medicine, Kanagawa, Japan; ^c^ Center for Dementia Care Research and Training in Tokyo, Tokyo, Japan; ^d^ Clinical Data & Biostatistics Department, Daiichi Sankyo Co., Ltd, Tokyo, Japan; ^e^ Medical Affairs Department, Daiichi Sankyo Co., Ltd, Tokyo, Japan

**Keywords:** Alzheimer's disease, behavior, cognition, language, memantine, outpatients

## Abstract

***Background:*** With the increase in the aging population, there is a pressing need to provide effective treatment options for individuals with Alzheimer's disease (AD). Memantine is an *N*-methyl-D-aspartate receptor antagonist used to treat AD in > 80 countries worldwide, and studies in the USA and Europe have shown it to be effective in improving language deficits; however, there are currently no data on language improvements in Japanese patients treated with memantine.

***Objectives:*** To clarify the efficacy and safety of memantine in Japanese outpatients with moderate to severe AD, using a pooled analysis of two multicenter randomized placebo-controlled trials, a phase 2 dose-finding study and a phase 3 study.

***Results:*** The final analysis comprised 633 patients (318 receiving memantine and 315 placebo). Memantine produced better outcomes in terms of Severe Impairment Battery-Japanese version, Clinician's Interview-Based Impression of Change plus-Japanese version, Behavioral Pathology in AD Rating Scale, and language scores, versus placebo. The overall incidence of adverse events and adverse reactions was similar between groups.

***Conclusion:*** In this pooled analysis of Japanese patients, memantine achieved better outcomes than placebo in terms of cognition, including attention, praxis, visuospatial ability and language, and behavioral and psychological symptoms, including activity disturbances and aggressiveness.

## 1. Introduction

In recent years, growth in the aging population has been observed in many countries and is progressing rapidly in Japan in particular. Because of this, the incidence of dementia–in particular, Alzheimer's disease (AD)–is rising rapidly, so there is a pressing need to provide effective treatment options for individuals with AD. In > 80 countries worldwide, memantine has been used to treat patients with AD (predominantly moderate-to-severe AD) for over 10 years, and its efficacy and safety have been reported in clinical trials and meta-analyses [1-3]. Memantine was approved for the treatment of moderate-to-severe AD in Japan in 2011. It is currently the only NMDA receptor antagonist for the treatment of AD [4]. A late Phase II dose-finding study in Japan demonstrated dose-responsiveness at 24 weeks in the Japanese version of the Severe Impairment Battery (SIB-J) [5,6]. A Phase III study, also conducted in Japan, showed a statistically significant change in SIB-J score compared with placebo. Regarding the variable the Clinician's Interview-Based Impression of Change plus-Japanese version (CIBIC plus-J) [7], the memantine group showed a lesser degree of worsening at week 24 compared with those receiving placebo, although the difference was not statistically significant [8]. Studies in the US and Europe have shown memantine to be effective in language improvement based on the SIB-Language (SIB-L) scale [9,10] and in behavioral improvement based on the 12-item Neuropsychiatric Inventory (NPI) [11]. However, there are no data on language improvements in Japanese patients treated with memantine and, given the differences in lifestyle and AD prevalence between Japan and Western countries, it remains unknown whether the results of clinical studies conducted outside Japan can be extrapolated to Japanese patients with AD. Taking into consideration the heterogeneity of AD, analysis of a large population could reveal information about the clinical effects of memantine on language ability in Japanese patients. Thus far, no analysis has been performed with a large population of Japanese subjects to evaluate the effects of memantine on behavioral improvement.

The objective of our study, therefore, was to clarify the efficacy–in terms of cognition, language, communication, and behavior–and safety of memantine in AD patients. To achieve this objective, we performed a pooled analysis of two previously published, multicenter randomized double-blind, placebo-controlled trials (Phase II and Phase III) [5,8] conducted in Japanese outpatients. We assessed the Behavioral Pathology in Alzheimer's Disease Rating Scale (BEHAVE-AD) [12], a subscale of CIBIC plus-J that was used in the Phase II and III studies, to analyze the effects of memantine on behavioral improvement. We also assessed the SIB-J to evaluate cognitive function [6], and the SIB-L to evaluate language [10]. The rationale for performing a pooled analysis was that it provided greater statistical power for detecting significant differences in these end points between memantine and placebo, providing confirmation of the results presented in the original reports.

## 2. Patients and methods

**Figure 1.**
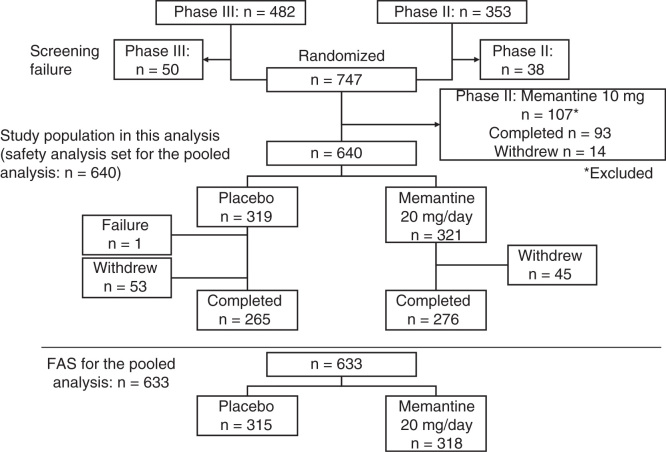
**Patient disposition in the two studies combined [5,8].** Seven patients were excluded from the full analysis set because of a lack of post-baseline efficacy data. Of these seven patients, three patients in each memantine group did not undergo post-baseline assessments because of eligibility violations or data for the primary efficacy endpoints (Severe Impairment Battery-Language-Japanese and Clinician's Interview-Based Impression of Change plus-Japanese) were missing, and one patient in the placebo group was suspected of having Creutzfeldt–Jacob disease, which was verified by genetic analysis, and was withdrawn from the study before the postbaseline evaluations.

The data for our pooled analysis were derived from a Phase II dose-finding study [5] and a Phase III study [8] of memantine in Japanese outpatients with moderate-to-severe AD. Because the studies had a similar design, unified analysis was possible. Table 1 provides an overview of each study, and Figure 1 provides the patient flow from the two studies combined. Both studies were conducted in accordance with Good Clinical Practice. Written voluntary consent regarding participation in the study was obtained from the patient and his or her legal representative. In instances where the patient was not capable of giving consent, or where consent was obtained orally and not in writing, written consent was obtained from the legal representative only.

**Table 1.** **Overview of the two studies [5,8].**

		**Phase II study**			**Phase III study**	
Diagnosis		Probable Alzheimer's disease according to NINCDS-ADRDA and DSM-IV				
Major inclusion criteria		Age ≥ 50 years MMSE score ≥ 5 and ≤ 14 FAST stage ≥ 6a and ≤ 7a				
Major exclusion criteria		Modified HIS ≥ 5 Major depression according to DSM-IV Comorbid other dementia with AD Comorbid other severe neurological disorder				
		Comorbid severe agitation			Comorbid other severe psychiatric disorder	
Duration (weeks)		24				
Design		Randomized, double-blind, placebo-controlled				
Primary end point		SIB-J, ADCS ADL-J			SIB-J, modified CIBIC plus-J	
		Memantine low	Memantine high	Placebo	Memantine	Placebo
No. of patients		107	100	107	218	208
Memantine treatment		10 mg	20 mg	–	20 mg	–
Sex	Male (%)	32.7	26.0	29.0	36.2	35.1
	Female (%)	67.3	74.0	71.0	63.8	64.9
Age (years)	Mean	73.2	73.2	73.6	74.4	74.9
MMSE score	Mean ± SD	9.8 ± 3.3	10.1 ± 2.7	10.4 ± 2.9	10.1 ± 3.0	9.6 ± 3.0
FAST stage*	Mean ± SD	2.8 ± 1.4	2.7 ± 1.4	2.5 ± 1.3	2.5 ± 1.2	2.5 ± 1.4

Fisher's exact test was used for sex and MMSE (category). The *t* test was used for age, body weight, MMSE, FAST and SIB-J. According to the DSM-IV criteria [13], patients should exhibit memory impairments plus at least one of aphasia, apraxia, agnosia and executive dysfunction. These cognitive deficits must progress slowly, persist, and be characterized by social/occupational dysfunction. These deficits must not be caused by other central nervous system diseases, systemic diseases known to cause dementia, induced psychotic disorder, delirium, or other major diseases. The NINCDS-ADRDA diagnostic criteria for probable AD [14] include dementia identified by clinical/neuropsychological tests, deficits in at least two cognitive regions, a progressive decline in memory and/or other cognitive functions, no disturbances in consciousness, onset of symptoms between 40 and 90 years of age, and the absence of systemic diseases or other brain diseases known to cause progressive memory/cognitive disorders. Probable diagnosis of AD may be supported by the presence of progressive aphasia, apraxia, or agnosia; impaired activities of daily living; family history of similar disorders; and clinical tests (e.g., spinal fluid tests, electroencephalography and computed tomography). *FAST: 6a = 1, 6b = 2, 6c = 3, 6d = 4, 6e = 5, 7a = 6. AD: Alzheimer's disease; ADCS-ADL-J: Alzheimer's Disease Cooperative Study-Activities of Daily Living Inventory [6]; CIBIC plus-J: Clinician's Interview-Based Impression of Change plus-Japanese version [7]; DSM-IV: Diagnostic and Statistical Manual of Mental Disorders, Fourth Edition [13]; FAST: Functional Assessment Staging of Alzheimer's disease [16]; MMSE: Mini-Mental State Examination [15]; Modified HIS: Modified Hachinski ischemic score [29]; NINCDS-ADRDA: National Institute of Neurological and Communicative Disorders and Stroke-Alzheimer's Disease and Related Disorders Association [14]; SIB-J: Severe Impairment Battery-Japanese version [6].

FAS: Full analysis set.

### 2.1 Study design

Fifty-three Japanese institutions participated in the Phase II study [5] and 74 participated in the Phase III study [8]. Both studies in our pooled analysis used a multicenter, double-blind, placebo-controlled, parallel-group design. The studies included a 4-week baseline observation period, during which patients received placebo once daily after breakfast. The double-blind period was 24 weeks in both studies, during which patients received memantine hydrochloride or placebo once daily after breakfast. The initial dose of memantine was 5 mg/day for 1 week, and the dose was then increased by 5 mg per week. The maintenance dose was 10 mg/day or 20 mg/day for the Phase II study, and 20 mg/day for the Phase III study. A total of 747 patients participated in the Phase II and Phase III studies (315 patients and 432 patients, respectively). Patients treated with memantine 10 mg/day in the Phase II study were excluded from the pooled analysis. Therefore, 321 patients treated with memantine 20 mg/day and 319 patients administered placebo were included in this pooled analysis. Patients and their caregivers visited the outpatient clinic once every 4 weeks and were evaluated for efficacy at the start of the double-blind period and at weeks 4, 12 and 24. Safety assessments took place seven times in total (at the predosing visit and weeks 4, 8, 12, 16, 20 and 24). Patients were followed up for 4 weeks after completion of the double-blind period or discontinuation of treatment to monitor adverse events (AEs). Concomitant use of any investigational drugs other than memantine, cholinesterase inhibitors (e.g., donepezil hydrochloride), NMDA receptor antagonists (e.g., ketamine), antiepileptic drugs, antiparkinsonian drugs, thiazide diuretics, or centrally acting muscle relaxants was prohibited. Concomitant use of hypnotics, sedatives, tranquilizers, or antipsychotics was prohibited in principle, but the use of brotizolam, rilmazafone, or lormetazepam was permitted only when necessary. The use of tiapride was allowed only when necessary or in a constant dosage regimen and within the normal dosage range (≤ 150 mg/day).

Regarding short-term in-hospital stays, patients were not allowed to receive short-term stays within 3 weeks of every efficacy evaluation (i.e., at the start of the double-blind period and weeks 4, 12 and 24) in the Phase II study. In the Phase III study, patients were not allowed to receive short-term stays at the start of the double-blind period and within the 3 weeks before the efficacy evaluation at week 24. The duration of short-term stays during the 4-week periods between the visits to the outpatient clinic for evaluation was not allowed to exceed six nights. When patients were already receiving rehabilitation such as long-term care services, this remained unchanged throughout the study period.

### 2.2 Patient population

Patient eligibility criteria included the following:

- Age ≥ 50 years
- Outpatient
- AD diagnosis according to the Diagnostic and Statistical Manual of Mental Disorders IV [13], or probable AD according to National Institute of Neurological and Communicative Disorders and Stroke-Alzheimer's Disease and Related Disorders Association criteria [14]
- Magnetic resonance imaging or computed tomography findings that were compatible with a diagnosis of AD and ruled out structural causes of dementia such as normal pressure hydrocephalus
- Mini-Mental State Examination (MMSE) score [15] of 5 – 14 at the start of treatment with placebo before the double-blind period (i.e., the observation period) and at the start of the double-blind period
- Moderate-to-severe AD according to the Functional Assessment Staging of Alzheimer's disease (FAST) [16], that is, stages ≥ 6a and ≤ 7a, at the start of treatment with placebo in the observation period

All participants must have been cared for by the same caregiver for > 3 days in a week throughout the study period. During the efficacy evaluation, the same caregiver had to be present with the patient.

Major exclusion criteria were as follows: cases complicated with dementia other than AD, a serious neurological disease or severe psychiatric disorder other than AD, and a history of treatment with memantine. Individuals who were planning to move into a nursing home or other care facility during the study period were also excluded.

### 2.3 Outcome measures

Efficacy measures used in this pooled analysis were as follows: SIB-J (cognitive function) [6], CIBIC plus-J (global assessment) [7,17], and the BEHAVE-AD (behavioral and psychological symptoms) [12]. In addition, we examined the SIB-L-J (a subscale of the SIB-J) in this analysis. The SIB-J is the Japanese version of the SIB [18,19]. The SIB was developed for evaluation of patients with severe cognitive impairment. The SIB-J consists of 40 items and has scores ranging from 0 to 100 points. The SIB-L consists of 21 items related to the Broca's area in the frontal lobe of the brain (relating to naming, reading, writing and repetition) among a total of 51 SIB items and has a maximum score of 41 points. A lower score indicates greater severity in language impairment. Consistent with the high variance of SIB-L scores within the three MMSE severity groups, the Pearson correlation coefficients between SIB-L and MMSE scores were low for the group with MMSE scores < 5 and moderate for the groups with MMSE scores 6 – 9 and 10 – 14. Additionally, the Pearson correlation coefficient for SIB-L with SIB was high, indicating that the SIB-L scale maintains the sensitivity of the complete SIB scale. The correlation was weaker between the nonlanguage SIB items and SIB-L items, but there were moderate correlations between SIB-L and level of functioning, as measured by the FAST and the Alzheimer's Disease Cooperative Study-Activities of Daily Living Inventory [10].

Stratified analysis of SIB-J data was performed according to the nine subdomains of the SIB-J at week 24 in both studies. The CIBIC plus-J [7,17] consists of Disability Assessment for Dementia or FAST to evaluate activities of daily living, the BEHAVE-AD [12] to evaluate behavioral and psychological symptoms of dementia, and the Mental Function Impairment Scale to evaluate core symptoms [20].

In addition to the assessments made using these scales, the absolute risk reduction (ARR) and number needed to treat (NNT) were determined as further indicators of treatment response. The ARR is the difference in responder rate between treatment groups, and NTT indicates the number of patients that needed to be treated to have one more patient with a defined treatment response [9].

### 2.4 Safety

Safety was assessed using the pooled incidence of AEs and adverse reactions. All AEs observed during the double-blind period and follow-up period of the Phase II and Phase III studies were examined with regard to the type, severity, time of onset, therapeutic measures and clinical course, and a possible causal relationship with memantine. In addition, general physical examinations, electrocardiograms, blood pressure and pulse rate, ophthalmologic examinations, and general laboratory tests were performed. The AEs were coded using the Japanese version of Medical Dictionary for Regulatory Activities version 11.1 preferred terms.

### 2.5 Statistical analysis

The efficacy analysis was performed for the full analysis set (FAS; Figure 1) using the same FASs as those used in the Phase II and Phase III studies [5,8]. The FAS is defined as the set of subjects that is as close as possible to the ideal based on the intention-to-treat principle [21]. The FAS excluded patients with eligibility violations, failure to take at least one dose of trial medication, and patients missing all postbaseline primary efficacy measurements (the latter exclusion criterion was applied only in the Phase III study). The safety analysis set included all patients enrolled in both studies.

Efficacy data at week 24 were analyzed by observed case (OC) and last observation carried forward (LOCF) analyses. The OC analysis was performed in cases available for evaluation at week 24, and the LOCF analysis was performed in cases available for evaluation at week 24 or using the most recently available data where data were missing at week 24.

For the SIB-J and BEHAVE-AD, changes in scores between the start of drug administration and each evaluation were compared using the Wilcoxon Rank-sum test (Wilcoxon test). For the analysis of CIBIC plus-J, scores at each evaluation were compared using the Mantel test. For proportions, comparisons were performed using Fisher's exact test or the χ^2^ test.

All analyses were performed using SAS® System Release 9.1.3 and 9.2 (SAS Institute, Cary, NC, USA). The significance level was set as 0.05 (two-sided).

## 3. Results

### 3.1 Study population

Of the 640 patients in this pooled analysis (safety analysis set for the pooled analysis), 321 were receiving memantine 20 mg/day and 319 were receiving placebo. There were no statistically significant differences between treatment groups in this pooled analysis in terms of baseline demographics (Table 2). Of the 640 patients enrolled in the double-blind period, the FAS comprised 633 patients (315 receiving placebo and 318 receiving memantine 20 mg/day) because of the exclusion of 7 patients who lacked postbaseline efficacy measurements. Of these seven patients, three patients in each memantine group did not undergo postbaseline assessments because of eligibility violations or data for the primary efficacy end points (SIB-J and CIBIC plus-J) were missing, and one patient in the placebo group was suspected of having Creutzfeldt–Jacob disease, which was verified by genetic analysis, and the patient was withdrawn from the study before the postbaseline evaluations. No patient was excluded for the reason of failure to take at least one dose of the trial medication (Figure 1).

**Table 2.** **Baseline patient demographics (full analysis set).**

		**Memantine** **(n = 318)**	**Placebo** **(n = 315)**	**Total** **(n = 633)**	p-value*
Sex (no. of patients [%])	Male	105 (33%)	104 (33%)	209 (33%)	1.0000
	Female	213 (67%)	211 (67%)	424 (67%)	
Age (years)	Mean ± SD	74.0 ± 8.9	74.5 ± 8.6	74.2 ± 8.8	0.4919
	Median	75.0	76.0	76.0	
	Minimum, maximum	50, 99	51, 94	50, 99	
Body weight (kg)	Mean ± SD	50.55 ± 9.41	50.11 ± 9.55	50.33 ± 9.47	0.5593
	Median	50.0	50.0	50.0	
	Minimum, maximum	28.4, 87.0	28.5, 79.7	28.4, 87.0	
MMSE score	Mean ± SD	10.09 ± 2.94	9.90 ± 2.96	9.99 ± 2.950	0.4109
	Median	10.5	10.0	10.0	
	Minimum, maximum	5, 14	5, 14	5, 14	
	No. of patients (%) with a score of 5 – 9	126 (39.6%)	129 (41%)	255 (40.3%)	0.7464
	No. of patients (%) with a score of 10 – 14	192 (60.4%)	186 (59.0%)	378 (59.7%)	
FAST stage	Mean ± SD	2.55 ± 1.30	2.49 ± 1.34	2.52 ± 1.32	0.579
	Median	2.0	2.0	2.0	
	Minimum, maximum	1, 6	1, 6	1, 6	
SIB-J score	No. of patients	318	313	631	0.5041
	Mean ± SD	71.86 ± 17.34	70.91 ± 18.40	71.39 ± 17.86	
	Median	75.0	76.0	76.0	
	Minimum, maximum	5, 97	5, 97	5, 97	

*Fisher's exact test was used for sex and MMSE (category). The *t*-test was used for age, body weight, MMSE, FAST and SIB-J. FAST: Functional Assessment Staging of Alzheimer's disease [16]; MMSE: Mini-Mental State Examination [15]; S.D.: Standard deviation; SIB-J: Severe Impairment Battery-Japanese version [6].

### 3.2 Cognitive function

**Figure 2.**
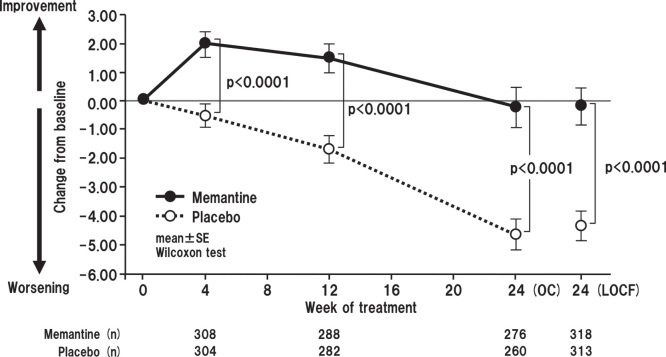
**Time course of change in total Severe Impairment Battery-Japanese version scores for OC (full analysis set) and change from baseline to week 24 with LOCF.** The difference between the FAS (633 patients; memantine, n = 618; placebo, n = 615) and LOCF (631 patients) is due to a lack of baseline data in two patients.

FAS: Full analysis set; LOCF: Last observation carried forward; OC: Observed cases; SE: Standard error.

**Figure 3.**
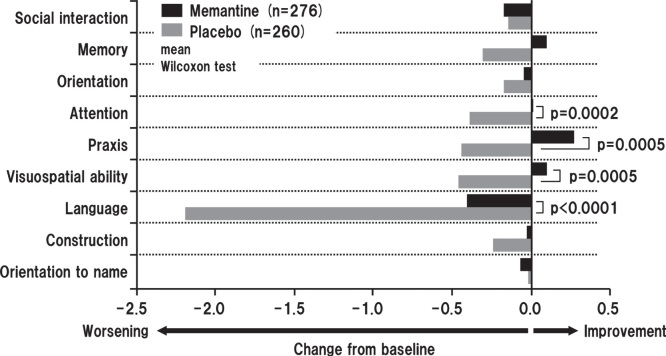
**Summary statistics of domain-specific changes in Severe Impairment Battery-Japanese version scores from baseline to week 24 (full analysis set, last observation carried forward analysis).** The difference between the FAS (633 patients; memantine, n = 618; placebo, n = 615) and LOCF (631 patients) is due to a lack of baseline data in two patients.

FAS: Full analysis set; LOCF: Last observation carried forward.

**Figure 4.**
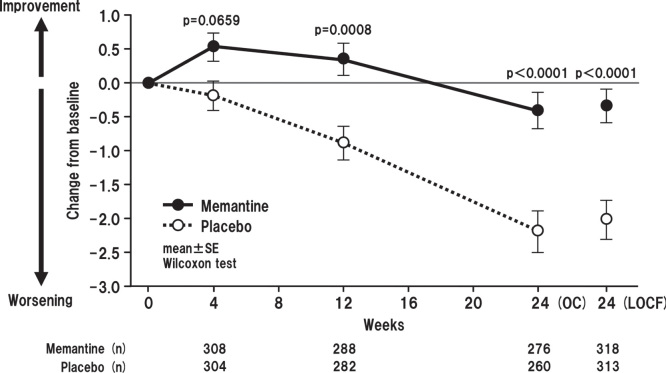
**Time course of mean changes from baseline in Severe Impairment Battery Language-Japanese version scores for OC (full analysis set) and change from baseline to week 24 with LOCF.** The difference between the FAS (633 patients; memantine, n = 618; placebo, n = 615) and LOCF (631 patients) is due to a lack of baseline data in two patients.

FAS: Full analysis set; LOCF: Last observation carried forward; OC: Observed cases; SE: Standard error.

Memantine produced statistically significantly better outcomes in SIB-J compared with placebo at each postbaseline evaluation (i.e., weeks 4, 12 and 24) in the OC analysis and at week 24 in the LOCF analysis. The mean differences in SIB-J score from baseline for the memantine and placebo groups in the OC analyses were 1.95 versus -0.45 (p < 0.0001; all p values presented herein are nominal p values), 1.51 versus -1.73 (p < 0.0001), and -0.34 versus -4.70 (p < 0.0001) for weeks 4, 12 and 24, respectively. In the LOCF analysis, mean differences in SIB-J scores were -0.25 for the memantine group and -4.38 in the placebo group (p < 0.0001) (Wilcoxon test) (Figure 2). When examining domain-specific score changes, mean differences from baseline were statistically significant for attention, praxis, visuospatial ability and language in both the OC (p = 0.0003, p = 0067, p = 0.0003, p < 0.0001, respectively) and the LOCF analyses (p = 0.0002, p = 0.0005, p = 0.0005, p < 0.0001, respectively) (Figure 3). Regarding changes in SIB-L-J scores, the memantine group showed a significantly better outcome at weeks 12 and 24 in the OC analysis and at week 24 in the LOCF analysis (Figure 4).

**Figure 5.**
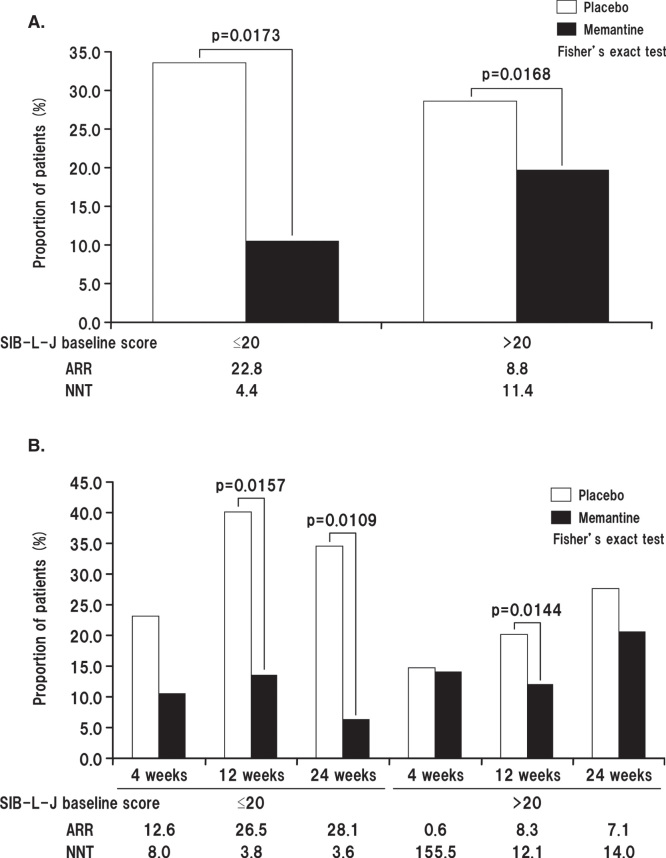
**A.** Proportion of patients showing worsening of Severe Impairment Battery-Language-Japanese version (SIB-J) scores at week 24 (full analysis set, last observation carried forward analysis) stratified by SIB-L-J baseline score (≤ 20 or > 20). **B.** Time course of changes in the proportion of patients with worsening in SIB-J score stratified by SIB-L-J baseline score (≤ 20 or > 20).

ARR: Absolute risk ratio; NNT: Number needed to treat.

The SIB-L-J scores were examined by stratifying patients based on their baseline SIB-L-J scores (≤ 20 or > 20). We assumed that the SIB-L-J score was worsened if the score was decreased by ≥ 3.7, a cut-off value used previously [9,10]. As a result, the SIB-L-J score was worsened in 33.3% of patients in the placebo group and 10.5% of patients in the memantine group. Among patients with baseline SIB-L-J scores ≤ 20 at week 24 (LOCF analysis), significantly fewer patients in the memantine group than in the placebo group showed worsening of SIB-L-J scores (p = 0.0173). Among patients with baseline SIB-L-J scores > 20, the SIB-L-J score was worsened in 28.4% of patients in the placebo group and 19.6% in the memantine group, again indicating a significantly lower incidence of score worsening in patients treated with memantine (p = 0.0168) (Figure 5A). At week 24 (LOCF analysis), the NNT for patients with at least no worsening in language performance was 3.6 patients (those with baseline SIB-L-J ≤ 20) and 14.0 patients (those with baseline SIB-L-J > 20) (Figure 5B).

### 3.3 Global assessment and behavioral and psychological symptoms

**Figure 6.**
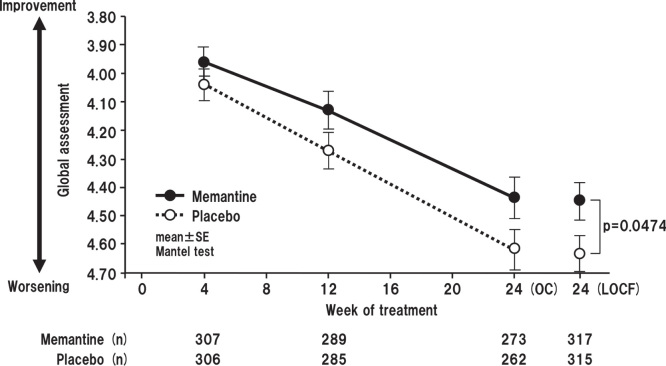
**Time course of global assessment score based on the Clinician's Interview-Based Impression of Change plus-Japanese (CIBIC plus-J) for OC and change from baseline to week 24 with LOCF.** The difference between the FAS (633 patients; memantine, n = 618; placebo, n = 615) and LOCF (632 patients) is due to a lack of baseline data in one patient.

FAS: Full analysis set; LOCF: Last observation carried forward; OC: Observed cases; SE: Standard error.

Memantine produced statistically significantly less worsening compared with placebo in terms of the CIBIC plus-J scores at week 24 in the LOCF analysis (mean values for memantine vs placebo 4.45 vs 4.64; p = 0.0474). In the OC analysis, the comparison was in favor of memantine, but did not reach the level of statistical significance (mean values for memantine vs placebo 3.96 vs 4.04, p = 0.2810; 4.13 vs 4.27, p = 0.1202; and 4.44 vs 4.62, p = 0.0843, at weeks 4, 12 and 24, respectively) (Figure 6).

**Figure 7.**
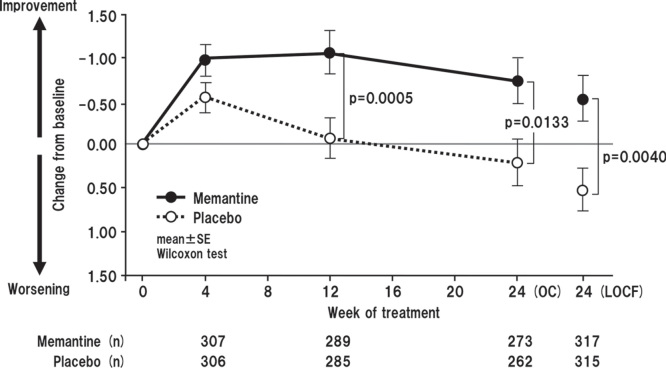
**Time course of change in total Behavioral Pathology in Alzheimer's Disease Rating Scale score for OC and change from baseline to week 24 with LOCF.** The difference between the FAS (633 patients; memantine, n = 618; placebo, n = 615) and LOCF (632 patients) is due to a lack of baseline data in one patient.

FAS: Full analysis set; LOCF: Last observation carried forward; OC: Observed cases; SE: Standard error.

In the analysis of changes in BEHAVE-AD scores, memantine showed statistically significant improvements compared with placebo at weeks 12 and 24 in the OC analysis, and in the LOCF analysis. Mean change from baseline for memantine versus placebo was -1.06 versus -0.07 (p = 0.0005), and -0.73 versus 0.21 (p = 0.0133), at weeks 12 and 24 in the OC analysis, respectively. In the LOCF analysis, the changes in score in the memantine and placebo groups were -0.52 and 0.52, respectively (p = 0. 0040) (Wilcoxon test) (Figure 7).

**Figure 8.**
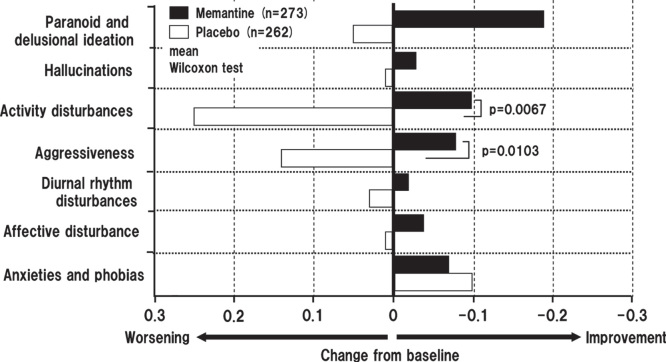
**Summary statistics of domain-specific score changes in Behavioral Pathology in Alzheimer's Disease Rating Scale from baseline to week 24 (last observation carried forward analysis).**

Using the week 24 BEHAVE-AD data, further analysis was carried out to stratify results according to the seven subdomains of BEHAVE-AD scale. For both the OC and the LOCF analyses, the differences were found to be statistically significant for the domains ‘activity disturbances' and ‘aggressiveness'. The mean differences from baseline for activity disturbances and aggressiveness were p = 0.0248 and p = 0.0227, respectively, in the OC analysis, and p = 0.0067 and p = 0.0103, respectively, in the LOCF analysis (Wilcoxon test) (Figure 8).

**Figure 9.**
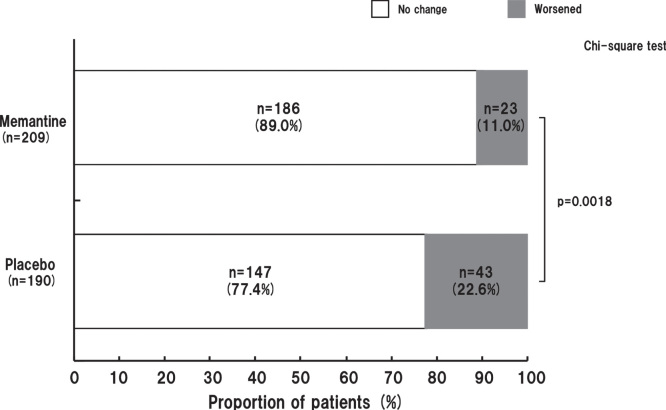
**The occurrence of new symptoms of aggressiveness in patients without aggressiveness at baseline.**

Efficacy data were also classified in the BEHAVE-AD domains according to the presence/absence of symptoms at study initiation. Memantine treatment exhibited a significantly better suppressing effect on the occurrence of new aggressiveness compared with placebo in patients without aggressiveness at study initiation (OC analysis p = 0.0063; LOCF analysis p = 0.0018 (χ^2^ test) (Figure 9).

### 3.4 Safety

The overall incidence of AEs was similar between the memantine and placebo groups (78.5 vs 76.8%). AEs with an incidence ≥ 5% in the memantine and placebo groups, respectively, were constipation (11.5 vs 10.3%), nasopharyngitis (14.3 vs 16.9%), fall (9.7 vs 10.3%), contusion (5.6 vs 6.3%), and insomnia (5.6 vs 5.0%) (Table 3).

**Table 3.** Adverse events with an incidence ≥ 5% in either treatment group*.

	**Memantine** **(n = 321)**		**Placebo** **(n = 319)**	
	**n**	**%**	**n**	**%**
Patients with adverse events	252	78.5	245	76.8
Constipation	37	11.5	33	10.3
Diarrhea	14	4.4	18	5.6
Vomiting	10	3.1	17	5.3
Nasopharyngitis	46	14.3	54	16.9
Fall	31	9.7	33	10.3
Contusion	18	5.6	20	6.3
Dementia Alzheimer's type^‡^	9	2.8	20	6.3
Insomnia	18	5.6	16	5.0

*Adverse events were coded according to preferred term using the Japanese version of the Medical Dictionary for Regulatory Activities version 11.1. ^‡^Corresponds to worsening of Alzheimer's disease.

## 4. Discussion

This pooled analysis demonstrated that memantine was statistically significantly superior to placebo as assessed by CIBIC plus-J scores in Japanese AD patients, similar to the findings of studies conducted in other countries [3]. Furthermore, the pooled analysis using the BEHAVE-AD–a subscale of the CIBIC plus-J–showed that memantine was also associated with less worsening of behavioral symptoms in Japanese AD patients compared with placebo. This pooled analysis also examined the subscales of the SIB-J, finding that memantine was associated with less worsening of language ability, visuospatial cognition, attention and praxis compared with placebo, again similar to the results of studies conducted overseas [1,2,22].

Language impairment is an important problem in AD. The SIB-L is a fast and easily administered scale that can be used for assessing language impairment and the effects of treatment on the language performance of patients with moderate-to-severe AD. Therefore, we also performed assessments using the SIB-L [9]. The removal of three items from the SIB language subscale yielded the SIB-L scale, an efficient and reliable tool for language assessment in moderate-to-severe AD patients. This patient population displays high variance in language performance. The SIB-L scale seems ideal for the assessment of basic language abilities and the evaluation of effects of treatment on language abilities in patients with moderate-to-severe AD [10].

Analysis of the SIB-L-J in this study revealed that memantine was associated with less worsening of language function throughout the treatment period compared with placebo. The SIB-L scale is an index for language performance, and Ferris *et al.* [9] have demonstrated the effectiveness of memantine based on changes in this scale. Tocco *et al.* [23] recently conducted a meta-analysis that included the results of the Japanese Phase II study and the study by Ferris *et al.* [9]. Taken together, the results appear to indicate that memantine is effective in the reduction of worsening of language ability in Japanese patients.

A *post hoc* analysis of the effect of memantine on behavior in three large randomized studies has been reported [24]. The study found a significant improvement in agitation/aggression in the NPI subitem cluster. Agitation/aggression in the NPI corresponds to aggressiveness in the BEHAVE-AD subitem cluster, which was analyzed in this pooled analysis. Our analysis showed that memantine significantly improved aggression, consistent with the results of studies conducted in Western countries [11,25].

A potential limitation of our study relates to the information obtained from the caregivers on the status of the patient. In the two studies included in our analysis, various measures were taken to obtain sufficient information from caregivers. In Japan, the number of people using long-term care services is reported to be increasing every year along with the changes in the long-term care insurance environment [26]. This was reflected in our study, whereby the number of subjects using long-term care services in the Phase III study was approximately 1.3 times higher than in the Phase II study. According to a report on recent nursing care services in Japan, AD patients tend to use long-term care services, leading to decreased observation of the patients by their family caregivers [27]. The report also states that information on the functional status of the patient from each caregiver may be insufficient because the burden of long-term care is often shared among family members in Japan. Considering these points, for those patients in our study who used long-term care services, information from only one caregiver might have been insufficient to reflect actual changes in clinical symptoms for the evaluation of CIBIC plus-J.

Regarding the statistical analysis performed in this study, because this pooled analysis is regarded as exploratory in nature, no adjustments were made for multiple observations. Therefore, further studies are needed to confirm the findings.

Because the observation period in this study was 24 weeks, any longer-term effect is unknown. While the effect of memantine on MMSE scores in a long-term study (mean duration 798 days) in Japanese patients has been demonstrated [28], the long-term effect on other functions in patients with AD needs to be clarified in further studies.

## 5. Conclusions

The results of our pooled analysis show that memantine is potentially effective and is well tolerated in patients with moderate-to-severe AD, consistent with findings of studies from other countries. In particular, the findings suggest that memantine may be beneficial in terms of cognition, including attention, praxis, visuospatial ability and language. Memantine also demonstrated a beneficial effect on behavioral and psychological symptoms, including activity disturbances and aggressiveness. These symptoms are known to be associated with rapid disease progression, increased caregiver burden, early institutionalization, and increased cost of care, so strategies to manage these symptoms, particularly in light of the growth in the aging population, are of great importance. These findings will be useful for physicians, patients, and their caregivers, adding to knowledge about the use of memantine in the Japanese setting. Because the mechanism of action of memantine differs from that of AChEIs, memantine is expected to be effective in nonresponders or poor responders to AChEIs. Thus, memantine may be used as initial monotherapy and in patients with inadequate or progressively deteriorating responses to AChEIs. Memantine may also be used in patients who experience AChEI-related AEs. Therefore, memantine hydrochloride is a new treatment option for AD and is expected to expand the therapeutic options for patients with AD. Further studies of a larger scale and with a clearly prespecified primary end point are required to confirm the present findings.

## Declaration of interest

This pooled analysis was supported by Daiichi Sankyo Co., Ltd. K Shiosakai and D Matsui are employees of Daiichi Sankyo Co., Ltd. Y Nakamura has received personal fees from Daiichi Sankyo Co., Ltd; Novartis; Takeda; Ono Pharmaceutical Co., Ltd; Janssen Pharmaceuticals, Inc.; Meiji Seika Pharma Co., Ltd; Mochida Pharmaceutical Co., Ltd; GlaxoSmithKline; Pfizer; Shionogi & Co., Ltd; Eli Lilly; Boehringer-Ingelheim; Toyama Chemical Co., Ltd; Astellas Pharma, Inc.; Dainippon Sumitomo Pharma Co., Ltd; Eisai Co., Ltd; Mitsubishi Tanabe Pharma; and Otsuka Pharmaceutical Co., Ltd, not during the current study. S Kitamura has received personal fees from Daiichi Sankyo Co., Ltd, not during the current study. A Homma has received personal fees from Daiichi Sankyo Co., Ltd, during the conduct of the current study, and personal fees from Eisai Co., Ltd; Novartis; Ono Pharmaceutical Co., Ltd; Takeda; Mitsubishi Tanabe Pharma; Otsuka Pharmaceutical Co., Ltd; Johnson & Johnson; and Toyama Chemical Co., Ltd, not during the current study. In addition, A Homma has a patent issued for a dementia screening system.
